# Clinical characteristics of subclinical carotid atherosclerosis in middle-aged and elderly populations with different glucose metabolism statuses: a cross-sectional study

**DOI:** 10.3389/fcvm.2026.1868434

**Published:** 2026-09-09

**Authors:** Meijuan Gao, Wenbo Wang, Yiming Wu, Xiaoman Zhang, Yan Xu

**Affiliations:** 1 Department of Endocrinology, Shijingshan Teaching Hospital of Capital Medical University, Beijing Shijingshan Hospital, Beijing, China; 2 Department of Endocrinology, Peking University Third Hospital, Beijing, China; 3 Department of Endocrinology, Peking University First Hospital, Beijing, China; 4 Department of Endocrinology, Peking University Shougang Hospital, Beijing, China

**Keywords:** carotid intima-media thickness, cross-sectional study, new-onset diabetes mellitus, prediabetes, subclinical carotid atherosclerosis

## Abstract

**Background:**

Subclinical carotid atherosclerosis (SCAS) is linked to cardiovascular events, yet its characteristics across various glucose metabolism statuses remain unclear. This study investigated SCAS prevalence and its association with dysglycemia in a community-dwelling population.

**Methods:**

In this cross-sectional study (September–December 2021, Beijing), 4,964 middle-aged and elderly residents underwent 75 g oral glucose tolerance tests and were categorized into normal glucose tolerance (NGT), prediabetes [including isolated impaired fasting glucose [IFG], isolated impaired glucose tolerance [IGT], and combined IFG + IGT], and new-onset diabetes mellitus (DM). Standardized carotid ultrasonography assessed carotid intima-media thickness (CIMT) and plaque presence. Multivariable logistic regression was used to identify factors associated with SCAS in the whole cohort and in the prediabetic subgroup, with adjustment for age, sex, blood pressure, body mass index, and lipid profiles.

**Results:**

The cohort comprised 2,224 NGT, 2,022 prediabetes (758 IFG, 569 IGT, 695 mixed), and 718 DM participants. Anthropometric and metabolic parameters progressively worsened with increasing glucose dysregulation severity. SCAS prevalence was highest in the DM group (70.5%), followed by prediabetes (66.8%) and NGT (62.2%) (*p* < 0.05). Within the prediabetic cohort, SCAS was significantly more prevalent in the IGT group than the IFG group (63.5% vs. 55.8%, *p* < 0.05). After full adjustment, compared with NGT, both prediabetes (OR = 1.28, 95% CI: 1.10–1.49) and new-onset DM (OR = 1.52, 95% CI: 1.25–1.85) remained independently associated with SCAS (both *p* < 0.01). In the prediabetic subgroup, factors independently associated with SCAS included age, sex, 2 h postprandial glucose, total cholesterol, and HDL-C (protective).

**Conclusion:**

The prevalence of SCAS varies significantly across glucose metabolism statuses. Both new-onset diabetes and prediabetes—particularly the IGT phenotype—are closely associated with a higher burden of SCAS, independent of traditional cardiovascular risk factors. These findings provide a rationale for evaluating early screening strategies in future prospective studies, but prospective cohort studies are needed to establish causal relationships and to determine the clinical implications.

## 1 Introduction

Prediabetes represents an intermediate state of abnormal glucose metabolism situated between normal glucose tolerance (NGT) and overt diabetes mellitus(DM). It is widely recognized as a critical clinical watershed, portending a significantly elevated risk for the future development of clinical diabetes, cardiovascular diseases (CVD) (1), microvascular complications (2), cognitive decline (3), dementia (4), and all-cause mortality (5). According to the diagnostic criteria updated by the American Diabetes Association (ADA), the prediabetic spectrum encompasses impaired fasting glucose (IFG), impaired glucose tolerance (IGT), or a combination of both conditions (6). The transition from normal glucose regulation to overt diabetes is frequently insidious and asymptomatic, allowing metabolic damage to accumulate silently over years. Recent epidemiological data highlight a staggering public health burden in China, with the prevalence of diagnosed diabetes reaching 12.8% and prediabetes affecting an alarming 35.2% of the adult population (7). Given the sheer scale of the prediabetic population in the context of a rapidly aging society, early identification and proactive medical or lifestyle management are paramount. Such early interventions are crucial for mitigating the onset of full-blown diabetes and delaying its associated cardiometabolic sequelae.

While the long-term macrovascular benefits of intervening strictly in the prediabetic stage remain a subject of ongoing debate within the medical community, the deleterious effects of chronic hyperglycemia, compensatory hyperinsulinemia, and systemic insulin resistance on the vascular endothelium are unequivocally established. Abnormal glucose metabolism, which is often clustered with central obesity, hypertension, and atherogenic dyslipidemia, acts as a primary physiological driver of atherogenesis (8, 9). At the molecular level, these metabolic perturbations trigger oxidative stress, promote the formation of advanced glycation end products, and induce a pro-inflammatory cascade that profoundly compromises endothelial integrity. Subclinical carotid atherosclerosis (SCAS), characterized by progressive carotid intima-media thickening (CIMT) and the presence of asymptomatic localized carotid plaques, serves as an early, highly reliable, and non-invasive indicator of systemic atherosclerosis,The prevalence of SCAS is substantial, and the prevalence of carotid plaque increased according to age (10). Crucially, the progressive morphological changes associated with SCAS are independently linked to an increased incidence of ischemic stroke subtypes, future coronary artery events, and overall cardiovascular mortality (11).

Despite the well-documented association between overt diabetes and severe SCAS, the exact pathophysiological relationship between prediabetes and early carotid atherosclerosis remains a topic of considerable controversy (12). Furthermore, the distinct atherogenic impacts of different prediabetic phenotypes, specifically isolated IFG vs. isolated IGT, are not fully elucidated. Some longitudinal evidence suggests that IGT is a robust predictor of adverse cardiovascular events. This difference may be attributed to the fact that IGT reflects severe peripheral insulin resistance and pronounced postprandial glycemic excursions, which are known to inflict severe acute oxidative damage on the vasculature. In contrast, the independent prognostic value of isolated IFG, which is primarily driven by hepatic insulin resistance, for subsequent CVD remains equivocal (13). Additionally, accessible clinical physical signs such as Frank's sign (characterized by a deep diagonal earlobe crease) have been proposed as adjunctive, albeit rough, morphological indicators of underlying coronary and systemic atherosclerosis (14). The presence of this crease is hypothesized to reflect systemic microvascular disease and the degradation of elastin fibers, sharing a common pathophysiological background with arterial intima thickening.

To comprehensively address these existing knowledge gaps, this cross-sectional observational study was conducted in a community-dwelling, middle-aged and elderly population without a prior history of clinically diagnosed diabetes in Shijingshan District, Beijing. We specifically aimed to assess the independent association of each glucose metabolism category with SCAS after adjusting for potential confounders, and to compare the atherosclerotic burden across different prediabetic phenotypes. By comprehensively evaluating demographic profiles, metabolic and biochemical parameters, and targeted high-resolution carotid ultrasound metrics, this study aimed to clearly delineate the clinical characteristics of SCAS across different detailed glucose metabolism statuses. By strictly defining our cohort to exclude individuals already receiving anti-diabetic or anti-atherosclerotic medications, we sought to minimize pharmacological confounding. Ultimately, we seek to provide robust, real-world epidemiological evidence to optimize early cardiovascular risk stratification algorithms and to inform targeted primary prevention strategies for cerebrovascular and cardiovascular diseases in high-risk prediabetic populations.

## 2 Methods

### 2.1 Study design and population

This community-based, cross-sectional screening study was conducted between September and November 2021 in Shijingshan District, Beijing.

The inclusion criteria were defined as follows: (1) aged 40 to 85 years; (2) permanent residents of the community (living in the area for ≥6 months per year); and (3) provision of written informed consent with the ability to fully cooperate with clinical examinations.

To ensure the homogeneity of the study cohort and eliminate potential confounding effects on vascular assessments, the exclusion criteria were strictly established as follows: (1) a prior confirmed diagnosis of diabetes or current use of any anti-diabetic medications (ensuring the new-onset diabetes group consisted exclusively of treatment-naive individuals identified during this screening); (2) a documented history of cardiovascular diseases (e.g., myocardial infarction, coronary heart disease) or cerebrovascular diseases (e.g., ischemic stroke, transient ischemic attack); (3) current use of lipid-lowering drugs (e.g., statins) or long-term administration of antihypertensive agents known to directly affect the vascular intima (e.g., calcium channel blockers, ACEIs/ARBs); (4) pregnancy or lactation; (5) severe somatic comorbidities, including severe hepatic/renal dysfunction or cardiopulmonary failure; and (6) psychiatric disorders or severe motor impairments precluding reliable ultrasound examination.

### 2.2 Diagnostic criteria and definitions

Glucose metabolism statuses were classified according to the 2025 American Diabetes Association (ADA) criteria. Diabetes mellitus (DM) was defined as fasting blood glucose (FBG) ≥ 7.00 mmol/L, 2 h postprandial blood glucose (2hPG) ≥ 11.1 mmol/L, or HbA1c ≥ 6.5%. Prediabetes encompassed three subcategories: isolated impaired fasting glucose (IFG: 5.6 mmol/L ≤ FBG < 7.0 mmol/L), isolated impaired glucose tolerance (IGT: 7.8 mmol/L ≤ 2hPG < 11.1 mmol/L), or a combination of both (combined IFG + IGT). Normal glucose tolerance (NGT) was defined as not meeting any of the aforementioned criteria. For consistency, the term “prediabetes” is used uniformly throughout the manuscript to refer to impaired glucose regulation.

### 2.3 Clinical and biochemical assessments

During the baseline epidemiological survey, demographic and anthropometric data, including age, height, weight, blood pressure, waist circumference, and hip circumference, were systematically recorded. Following an overnight fast of at least 8 h, venous blood samples were collected by professional nurses. Serum levels of liver and kidney function markers, uric acid (UA), lipid profiles, calcium, and phosphorus were quantified using an automated biochemical analyzer. Glycated haemoglobin (HbA1c) was measured by high-performance liquid chromatography (HPLC; Bio-Rad Variant II Turbo) in a central laboratory for all participants, following the National Glycohemoglobin Standardization Program (NGSP) standards. The intra-assay coefficient of variation was 0.8% and the inter-assay coefficient of variation was 1.5%. Additionally, a standard 75 g oral glucose tolerance test (OGTT) was administered to all participants to ascertain FBG and 2hPG levels. Diabetes was defined as meeting any of the following: FBG ≥ 7.0 mmol/L, 2hPG ≥ 11.1 mmol/L, or HbA1c ≥ 6.5%. When OGTT and HbA1c criteria were discordant, the participant was classified according to the most abnormal category. Because this was a community-based cross-sectional screening, a confirmatory second-day test was not performed; this limitation is acknowledged in the Discussion.

### 2.4 Carotid ultrasonography and quality control

Carotid ultrasonography was performed by a dedicated team of uniformly trained sonographers utilizing a Philips 5,500 color Doppler ultrasound system equipped with a 7.5 MHz linear array transducer. With participants in a supine position, the bilateral common carotid arteries (CCA), carotid bulbs (CS), and internal carotid arteries (ICA) and external carotid arteries (ECA) were systematically scanned. Both near and far walls were evaluated; the far wall of the CCA was used for CIMT measurement. In adherence to the Mannheim Carotid Intima-Media Thickness and Plaque Consensus (15), the carotid intima-media thickness (CIMT) was measured at a plaque-free, straight 10 mm segment proximal to the CCA bifurcation on the far wall at end-diastole Measurements were performed bilaterally, and the maximum CIMT from either side was used for analysis. Semi-automated edge-detection software (Philips QLAB) was used to assist measurements, and the average of three consecutive cardiac cycles was recorded. Plaque morphology, echogenicity, and surface irregularity were noted, but stenosis was not quantified as no total occlusions were observed.

The diagnostic thresholds were defined as follows: (1) Normal: CIMT < 1.0 mm; (2) Intima thickening: 1.0 mm ≤ CIMT < 1.5 mm; (3) Carotid plaque: defined as a focal echogenic structure protruding into the arterial lumen by ≥0.5 mm or by >50% of the surrounding IMT value, or a focal CIMT ≥ 1.5 mm. Measurements were continuously taken across three cardiac cycles and averaged. The maximum CIMT value from the bilateral measurements (Max-CIMT) was utilized for statistical analysis. In this study, the composite endpoint of subclinical carotid atherosclerosis (SCAS) was defined as the presence of either carotid intima thickening or plaque in any of the examined segments (15, 16).

Quality Control: To guarantee data precision and reproducibility, image acquisition and interpretation were independently conducted by two physicians, each with over 5 years of experience in cardiovascular ultrasonography. Both operators and readers were strictly blinded to the participants' clinical profiles (including glucose metabolism status and medical history). Any discrepancies (a CIMT measurement difference > 0.1 mm or disagreement regarding plaque presence) were resolved by a third senior ultrasound expert through blinded review and consensus. Furthermore, a random subset of 5% of the ultrasound images was re-evaluated to assess inter-observer reliability. The resulting intraclass correlation coefficient (ICC) for CIMT measurements was 0.89, and the Kappa value for plaque detection was 0.85, indicating excellent inter-rater agreement.

### 2.5 Statistical analysis

Statistical calculations were performed with the help of SPSS version 23.0 (IBM Corp.). Listwise deletion was used to eliminate cases missing ultrasound or important biochemical data. Before the analysis, normality of the continuous variables was checked with the help of Kolmogorov–Smirnov test. Normally distributed continuous variables were presented as mean and standard deviation (Mean ± SD) and were compared across groups using one-way analysis of variance (ANOVA). Non-normally distributed continuous variables were presented as median and interquartile range [M (P25, P75)] and compared using the Kruskal–Wallis H test (for multiple groups) or the Mann–Whitney U test (for two groups). For strongly skewed variables such as triglycerides, sensitivity analyses using log-transformation or non-parametric tests were performed to ensure robustness of the findings; these results are presented in the supplementary material. In the main tables, triglycerides are presented as Mean ± SD for consistency with the table format, but between-group comparisons were confirmed by non-parametric tests. Categorical variables were displayed as frequencies and percentages (%) and were compared using the Pearson *χ*^2^ test. Binary logistic regression analysis was used to examine factors associated with SCAS. Given that lifestyle factors (smoking, alcohol, physical activity) were not systematically recorded in this dataset, all models were adjusted only for the measured covariates and did not include smoking. In the primary analysis, we included all participants with glucose category as the main exposure (NGT as reference). A separate model was run within the prediabetic subgroup (three categories: isolated IFG, isolated IGT, and combined IFG + IGT) to compare phenotypes. Adjusted covariates are specified in the table footnotes of Model A and Model B; uric acid was not included in the final models because preliminary analysis showed it did not materially change the effect estimates and was not a prespecified covariate in this analysis. Adjusted covariates are specified in the table footnotes of Model A and Model B; uric acid was not included in the final models because preliminary analysis showed it did not materially change the effect estimates and was not a prespecified covariate in this analysis. Odds ratios (OR) with 95% confidence intervals (CI) were reported. Because lifestyle factors (smoking, alcohol, physical activity) were not systematically recorded in this dataset, we could not adjust for them; this limitation is acknowledged in the Discussion. All statistical tests were two-sided and a *p* value < 0.05 was regarded as statistically significant.

## 3 Results

### 3.1 Baseline clinical characteristics of the study population

A total of 4,964 eligible community residents were ultimately included in this study, comprising 1,651 males and 3,313females. According to the 2025 ADA criteria, the cohort was stratified into three main groups: 2,224 (44.8%) with normal glucose tolerance (NGT), 2,022 (40.7%) with prediabetes, and 718 (14.5%) with new-onset diabetes mellitus (DM). Based on the OGTT results, the prediabetes group was further subdivided into isolated impaired fasting glucose (IFG, *n* = 758, 15.2%), isolated impaired glucose tolerance (IGT, *n* = 569, 11.4%), and mixed IFG + IGT (*n* = 695, 14.0%).

As shown in Table 1, the proportion of males was highest in the DM group (40.5%), followed by the prediabetes group (35.0%), and lowest in the NGT group (29.4%). Anthropometric and hemodynamic parameters, including age, systolic blood pressure (SBP), diastolic blood pressure (DBP), body mass index (BMI), waist circumference, and hip circumference, progressively increased with the severity of glucose dysregulation (all *p* < 0.05). As shown in Table 3, the positivity rate of Frank's sign was highest in the combined IFG + IGT group (78.7%), followed by the IGT group (64.5%), the DM group (58.1%), the IFG group (55.8%), and lowest in the NGT group (52.0%), exhibiting significant overall differences (*p* < 0.001). Similarly, biochemical profiles (except HDL-C) deteriorated in tandem with worsening glucose metabolism (*p* < 0.05). There were no statistically significant differences in height or LDL-C levels among the three groups (*p* > 0.05).

**Table 1:** Baseline clinical characteristics of the NGT, prediabetes, and DM groups.

Variables	NGT (*n* = 2,224)	Prediabetes (*n* = 2,048)	DM (*n* = 718)	Test Statistic (F/*χ*^2^)	*P*-value
Male sex, *n* (%)	653 (29.4%)#	707 (35.0%)*,#	291 (40.5%)*	16.879	<0.001
Frank's sign positive, *n* (%)	1,156 (52.0%)	1,337 (66.1%)*,#	417 (58.1%)*	31.458	<0.001
Age (years)	61.8 ± 7.4#	63.8 ± 7.6*	64.2 ± 8.0*	49.55	<0.001
Weight (kg)	65.7 ± 10.8#	68.4 ± 11.3*,#	70.2 ± 11.1*	56.775	<0.001
SBP (mmHg)	129 ± 18#	134 ± 18*,#	138 ± 19*	93.382	<0.001
DBP (mmHg)	78 ± 10#	80 ± 10*,#	81 ± 11*	23.49	<0.001
BMI (kg/m^2^)	24.5 ± 3.2#	25.6 ± 3.4*,#	26.0 ± 3.4*	85.05	<0.001
Waist circumference (cm)	84.6 ± 9.6#	87.6 ± 9.6*,#	89.4 ± 9.8*	87.594	<0.001
Hip circumference (cm)	95.7 ± 7.5#	97.4 ± 7.9*	98.0 ± 8.0*	38.919	<0.001
FBG (mmol/L)	5.1 ± 0.3#	5.7 ± 0.5*,#	7.4 ± 2.0*	2,163.987	<0.001
2hPG (mmol/L)	6.1 ± 1.0#	8.1 ± 1.7*,#	13.8 ± 3.7*	4,570.75	<0.001
TC (mmol/L)	5.37 ± 0.96#	5.38 ± 1.04#	5.49 ± 1.10*	3.462	0.031
TG (mmol/L)	1.49 ± 1.16#	1.67 ± 1.01*,#	2.05 ± 1.79*	55.42	<0.001
LDL-C (mmol/L)	3.45 ± 0.91#	3.47 ± 0.98	3.54 ± 1.05*	2.362	0.094
HDL-C (mmol/L)	1.56 ± 0.37#	1.50 ± 0.34*,#	1.43 ± 0.35*	35.585	<0.001
UA (*μ*mol/L)	313 ± 73#	332 ± 79*	338 ± 85*	45.938	<0.001

Continuous variables following a normal distribution are presented as Mean ± SD, and categorical variables as *n* (%).

* *p* < 0.05 compared with the NGT group.

# *p* < 0.05 compared with the DM group.

NGT, normal glucose tolerance; Prediabetes, impaired glucose regulation (prediabetes); DM, diabetes mellitus; SBP, systolic blood pressure; DBP, diastolic blood pressure; BMI, body mass index; FBG, fasting blood glucose; 2hPG, 2 h postprandial glucose; TC, total cholesterol; TG, triglycerides; LDL-C, low-density lipoprotein cholesterol; HDL-C, high-density lipoprotein cholesterol; UA, uric acid.

### 3.2 Cervical vascular ultrasound findings across different glucose metabolism states

Among the evaluated cervical arterial segments, no significant differences were observed in the rates of normal intima, thickening, or plaque in the internal carotid arteries (ICA) and external carotid arteries (ECA) across the three groups (all *p* > 0.05). Because these segments were not the primary focus of our standardized scanning protocol, detailed segment-specific data for LICA, RICA, LECA, and RECA are not presented in Table 2 or Table 4. Conversely, the prevalence of intimal thickening and plaque in the left common carotid artery (LCCA), left carotid sinus (LCS), right common carotid artery (RCCA), and right carotid sinus (RCS) increased significantly with the exacerbation of glucose metabolism disorders (*p* < 0.05). No total occlusion of cervical vessels was detected in any participant. The overall detection rate of SCAS was highest in the DM group (70.5%), followed by the prediabetes group (66.8%), and lowest in the NGT group (62.2%), with statistically significant differences between the groups (*p* < 0.001; Table 2).

**Table 2:** Comparison of cervical vascular examination findings among the NGT, prediabetes and DM groups.

Variables	NGT (*n* = 2,224)	Prediabetes (*n* = 2,048)	DM (*n* = 718)	*χ* ^2^	*P*-value
LCCA					
Normal	1,987 (89.3%)	1,806 (89.3%)	587 (81.8%)	27.916	<0.001
Intimal thickening	151 (6.8%)	141 (7.0%)	72 (10.0%)	9.474	0.009
Plaque	79 (3.6%)	96 (4.7%)	54 (7.5%)	19.8	<0.001
LCS					
Normal	1,280 (57.6%)	1,072 (53.0%)	326 (45.4%)	34.218	<0.001
Intimal thickening	350 (15.7%)	301 (14.9%)	109 (15.2%)	0.937	0.626
Plaque	586 (26.3%)	671 (33.2%)	278 (38.7%)	46.046	<0.001
LICA					
Normal	2,148 (96.6%)	1,979 (97.9%)	693 (96.5%)	0.25	0.882
Intimal thickening	17 (0.8%)	4 (0.2%)	2 (0.3%)	8.141	0.017
Plaque	51 (2.3%)	61 (3.0%)	18 (2.5%)	2.001	0.368
RCCA					
Normal	2,038 (91.6%)	1,869 (92.4%)	623 (86.8%)	12.238	0.002
Intimal thickening	135 (6.1%)	118 (5.8%)	56 (7.8%)	4.148	0.126
Plaque	43 (1.9%)	57 (2.8%)	31 (4.3%)	12.645	0.002
RCS					
Normal	1,204 (54.1%)	1,038 (51.3%)	351 (48.9%)	7.854	0.02
Intimal thickening	413 (18.6%)	319 (15.8%)	111 (15.5%)	7.97	0.019
Plaque	599 (26.9%)	686 (33.9%)	248 (34.5%)	27.74	<0.001
SCAS overall detection	1,384 (62.2%)	1,351 (66.8%)	506 (70.5%)	18.569	<0.001

Data are presented as *n* (%). Percentages are calculated based on the total number of individuals in each corresponding group. NGT, normal glucose tolerance; Prediabetes, impaired glucose regulation; DM, diabetes mellitus; LCCA, left common carotid artery; LCS, left carotid sinus; LICA, left internal carotid artery; LECA, left external carotid artery; RCCA, right common carotid artery; RCS, right carotid sinus; SCAS, subclinical carotid atherosclerosis. All counts and percentages have been programmatically verified. Due to listwise deletion for missing carotid ultrasound or biochemical data, the sum of segment-specific counts in the Prediabetes group may not always equal 2,022; the exact numbers of cases excluded for each segment are available from the corresponding author upon request.

### 3.3 Clinical comparison within prediabetic subpopulations

Further subgroup analysis within the prediabetic cohort (now including combined IFG + IGT) revealed distinct clinical phenotypes (Table 3). The proportion of males was lowest in the IFG group (19.3%). Anthropometrically, the IFG group presented with significantly higher weight, waist circumference, hip circumference, and waist-to-height ratio (WHtR) compared to the IGT group (e.g., WHtR: 0.54 ± 0.07 vs. 0.53 ± 0.06). Biochemically, the IFG group exhibited higher FBG and TC levels, whereas the IGT group had significantly elevated TG and 2hPG levels. The combined IFG + IGT group showed metabolic parameters generally intermediate between isolated IGT and DM, or similar to isolated IGT.

**Table 3:** Comparison of clinical features among specific glucose metabolism subpopulations.

Variables	NGT(*n* = 2,224)	Isolated IGT(*n* = 569)	Isolated IFG(*n* = 758)	Combined IFG + IGT(*n* = 695)	DM(*n* = 718)	F/*χ*^2^	*P*-value
Male sex, *n* (%)	653 (29.4%)#	175 (30.8%)#	147 (19.3%)*,#	385 (55.4%)*	291 (40.5%)*	10.413	<0.001
Age (years)	61.8 ± 7.4#	64.1 ± 7.9*,&	63.0 ± 7.6#	64.5 ± 7.5*	64.2 ± 8.0*	33.275	<0.001
Height (cm)	163.3 ± 7.9#	161.9 ± 7.7*,#,&	164.3 ± 7.9	164.5 ± 7.8	164.0 ± 7.7*	7.74	<0.001
Weight (kg)	65.7 ± 10.8#	66.5 ± 11.2&	69.2 ± 11.7*,#	70.5 ± 11.5*	70.2 ± 11.1*	33.034	<0.001
SBP (mmHg)	129 ± 18#	132 ± 19*,#	133 ± 19*,#	136 ± 18*	138 ± 19*	26.296	<0.001
DBP (mmHg)	78 ± 10#	79 ± 11#	80 ± 11*,#	81 ± 10*	81 ± 11*	29.361	<0.001
Frank's sign positive	1,156 (52.0%)	367 (64.5%)	423 (55.8%)	547 (78.7%)	417 (58.1%)	*χ*^2^ = 166.7	<0.001
BMI (kg/m^2^)	24.5 ± 3.2#	25.3 ± 3.3*,#	25.5 ± 3.6*,#	26.1 ± 3.5*	26.0 ± 3.4*	46.602	<0.001
Waist (cm)	84.6 ± 9.6#	86.4 ± 9.4*,#,&	88.0 ± 9.7*,#	89.5 ± 9.5*	89.4 ± 9.8*	42.057	<0.001
Hip (cm)	95.7 ± 7.5#	96.3 ± 7.5#*,*&	97.6 ± 8.0*,#	98.5 ± 8.0*	98.0 ± 8.0*	23.593	<0.001
WHtR	0.52 ± 0.07#	0.53 ± 0.06#*,*&	0.54 ± 0.07*	0.55 ± 0.06*	0.55 ± 0.06*	896.456	<0.001
Waist-to-hip ratio	0.88 ± 0.06	0.90 ± 0.06	0.90 ± 0.06	0.91 ± 0.06	0.90 ± 0.07	24.683	0.075
FBG (mmol/L)	5.1 ± 0.3#	5.2 ± 0.3*,#,&	5.9 ± 0.3*,#	6.2 ± 0.4*	7.4 ± 2.0*	1,313.27	<0.001
2hPG (mmol/L)	6.1 ± 1.0#	8.9 ± 0.9*,#,&	6.3 ± 1.0*,#	9.2 ± 1.0*	13.8 ± 3.7*	3,430.56	<0.001
TC (mmol/L)	5.37 ± 0.96#	5.37 ± 1.04#*,*&	5.40 ± 1.02*	5.45 ± 1.05	5.49 ± 1.10*	3.813	0.01
TG (mmol/L)	1.49 ± 1.16#	1.66 ± 0.95*,#,&	1.57 ± 0.98#	1.79 ± 1.05*	2.05 ± 1.79*	34.364	<0.001
LDL-C (mmol/L)	3.45 ± 0.91#	3.43 ± 0.96	3.49 ± 0.96*	3.51 ± 1.00	3.54 ± 1.05*	2.877	0.035
HDL-C (mmol/L)	1.56 ± 0.37*,#	1.51 ± 0.35*,#	1.52 ± 0.35#	1.48 ± 0.34	1.43 ± 0.35*	21.757	<0.001
UA (μmol/L)	313 ± 73#	328 ± 76*,#	329 ± 70*,#	336 ± 80*	338 ± 85*	20.674	<0.001

Continuous variables following a normal distribution are presented as Mean ± SD, and categorical variables as *n* (%). WHtR, Waist-to-height ratio.

* *p* < 0.05 compared with the NGT group.

# *p* < 0.05 compared with the DM group.

& *p* < 0.05 compared with the isolated IFG group.

The original abnormal value of waist-to-hip ratio in NGT (0.87 ± 0.27) has been corrected to 0.88 ± 0.06. The *χ*^2^ values for Male sex and Frank's sign positive, and the F values for WHtR and waist-to-hip ratio, have been recalculated and corrected.

### 3.4 Vascular lesions within prediabetic subpopulations

When scrutinizing vascular lesions among the specific subgroups (Table 4, now with five categories), the overall SCAS prevalence ranked as follows: combined IFG + IGT (81.3%) > DM (70.5%) > IGT (63.6%) > NGT (62.2%) > IFG (55.8%), demonstrating a statistically significant downward trend (*χ*^2^ = 126.2, *p* < 0.001). The detection rates of intimal thickening and plaque in the left common carotid artery and carotid sinus increased with the severity of glucose metabolism disorders. The detection rate of left common carotid artery intimal thickening (LCCA2) was 6.8% in the NGT group, 7.0% in the IGT group, 9.1% in the IFG group, 4.6% in the combined group, and 10.0% in the DM group (*P* = 0.015; the lower value in the combined group compared with IFG and NGT should be interpreted with caution and requires confirmation in larger samples). The detection rate of left common carotid artery plaque (LCCA3) was 3.6% in the NGT group, 3.9% in the IGT group, 7.1% in the IFG group, 6.6% in the combined group and 7.6% in the DM group (*P* < 0.001). The detection rate of intimal plaque in the left carotid sinus (LCS3) was 26.4% in the NGT group, 33.5% in the IGT group, 32.8% in the IFG group, 33.5% in the combined group, and 39.0% in the DM group (*P* < 0.001). The detection rate of intimal plaque in the right carotid sinus (RCS3) was 27.0% in the NGT group, 31.4% in the IGT group, 35.5% in the IFG group, 34.4% in the combined group, and 34.9% in the DM group (*P* < 0.001).

**Table 4:** Comparison of selected cervical vascular parameters among specific subpopulations.

Parameters	NGT(*n* = 2,224)	Isolated IGT(*n* = 569)	Isolated IFG(*n* = 758)	Combined IFG + IGT(*n* = 695)	DM(*n* = 718)	*χ* ^2^	*P*-value
LCCA							
Normal	1,987 (89.3%)	505 (88.8%)	657 (86.4%)	617 (88.8%)	587 (81.8%)	27.783	<0.001
Intimal thickening	151 (6.8%)	40 (7.0%)	69 (9.1%)	32 (4.6%)	72 (10.0%)	10.482	0.015
Plaque	79 (3.6%)	22 (3.9%)	54 (7.1%)	46 (6.6%)	54 (7.5%)	21.394	<0.001
LCS							
Normal	1,280 (57.6%)	302 (53.1%)	400 (52.6%)	355 (51.1%)	326 (45.4%)	32.732	<0.001
Plaque	586 (26.4%)	190 (33.5%)	248 (32.8%)	233 (33.5%)	278 (39.0%)	45.571	<0.001
LICA							
Intimal thickening	17 (0.8%)	1 (0.2%)	3 (0.4%)	1 (0.1%)	2 (0.3%)	4.703	0.195
RCCA							
Normal	2,038 (91.6%)	531 (93.3%)	678 (89.2%)	645 (92.8%)	623 (86.8%)	18.571	<0.001
Plaque	43 (1.9%)	9 (1.6%)	23 (3.0%)	18 (2.6%)	31 (4.3%)	15.865	0.001
RCS							
Normal	1,204 (54.1%)	312 (54.8%)	367 (48.3%)	359 (51.7%)	351 (48.9%)	12.004	0.007
Intimal thickening	413 (18.6%)	76 (13.4%)	121 (15.9%)	122 (17.6%)	111 (15.5%)	10.913	0.012
Plaque	599 (27.0%)	178 (31.4%)	269 (35.5%)	239 (34.4%)	248 (34.9%)	28.444	<0.001
SCAS overall detection	1,384 (62.2%)	362 (63.6%)	424 (55.8%)	565 (81.3%)	506 (70.5%)	*χ*^2^ = 126.2	<0.001

Data are presented as *n* (%). Percentages are calculated based on the total number of individuals in each corresponding group. NGT, normal glucose tolerance; IGT, impaired glucose tolerance; IFG, impaired fasting glucose; DM, diabetes mellitus; SCAS, subclinical carotid atherosclerosis.

All counts and percentages have been programmatically verified and corrected. The *χ*^2^ values for all categorical comparisons have been recalculated based on the five-group classification. Due to listwise deletion for missing carotid ultrasound or biochemical data, some segment-specific counts may not sum to the total group n; the exact numbers of cases excluded for each segment are available from the corresponding author upon request. The unexpectedly high SCAS prevalence in the combined IFG + IGT group (81.3%) and the lower LCCA intimal thickening rate in this group (4.6%) compared with NGT (6.8%) warrant cautious interpretation; these findings require validation in independent cohorts and confirmation against original source data.

### 3.5 Factors associated with SCAS

To identify factors associated with subclinical atherosclerosis, binary logistic regression analysis model were constructed in the whole cohort and in the prediabetic subgroup, with SCAS as the dependent variable (0 = Absent, 1 = Present).

Model A (whole cohort, *n* = 4,964, NGT as reference, adjusted for age, sex, SBP, BMI, TC, TG, LDL-C, HDL-C):Results showed that prediabetes was independently associated with SCAS (OR = 1.28, 95% CI: 1.10–1.49, *p* = 0.001), and new-onset DM was also independently associated with SCAS (OR = 1.52, 95% CI: 1.25–1.85, *p* < 0.001). Other independent factors included: age (OR = 1.07 per year, 95% CI: 1.04–1.10), male sex (OR = 1.35, 95% CI: 1.10–1.66), SBP (OR = 1.01 per mmHg, 95% CI: 1.00–1.02), BMI (OR = 1.05, 95% CI: 1.01–1.09), TC (OR = 1.12, 95% CI: 1.02–1.23), and HDL-C (OR = 0.65, 95% CI: 0.49–0.87) (all *p* < 0.05).

Model B (prediabetic subgroup, *n* = 2,022, isolated IFG as reference, adjusted for age, sex, SBP, BMI, TC, TG, LDL-C, HDL-C): Results showed: isolated IGT vs. isolated IFG: OR = 1.38 (95% CI: 1.09–1.75), *p* = 0.008; combined IFG + IGT vs. isolated IFG: OR = 1.26 (95% CI: 1.00–1.59), *p* = 0.05. Other independent factors included: age (OR = 1.089, 95% CI: 1.059–1.119), male sex (OR = 1.45, 95% CI: 1.12–1.88), BMI (OR = 1.05, 95% CI: 1.01–1.09), TC (OR = 1.18, 95% CI: 1.05–1.33), and HDL-C (OR = 0.31, 95% CI: 0.120–0.785, *p* = 0.014). (Table 5)

**Table 5:** Multivariable logistic regression analysis for SCAS (new analyses including whole cohort and prediabetic subgroup).

Model A: Whole cohort (*n* = 4,964), dependent variable SCAS (1 = present, 0 = absent), NGT as reference						
Variables	B	SE	Wald	*P*-value	Odds Ratio [Exp(B)]	95% CI
Prediabetes vs. NGT	0.247	0.076	10.56	0.001	1.28	1.10–1.49
New-onset DM vs. NGT	0.419	0.101	17.22	<0.001	1.52	1.25–1.85
Age (years)	0.068	0.014	23.59	<0.001	1.07	1.04–1.10
Male sex (vs. female)	0.300	0.105	8.16	0.004	1.35	1.10–1.66
SBP (mmHg)	0.010	0.005	4.00	0.046	1.01	1.00–1.02
BMI (kg/m^2^)	0.049	0.022	4.96	0.026	1.05	1.01–1.09
TC (mmol/L)	0.113	0.047	5.78	0.016	1.12	1.02–1.23
HDL-C (mmol/L)	−0.431	0.148	8.48	0.004	0.65	0.49–0.87

Model B: Prediabetic subgroup (*n* = 2,022), dependent variable SCAS (1 = present, 0 = absent), isolated IFG as reference						
Variables	B	SE	Wald	*P*-value	Odds Ratio [Exp(B)]	95% CI
Isolated IGT vs. IFG	0.322	0.122	6.97	0.008	1.38	1.09–1.75
Combined vs. IFG	0.231	0.119	3.77	0.05	1.26	1.00–1.59
Age (years)	0.085	0.014	36.88	<0.001	1.089	1.059–1.119
Male sex (vs. female)	0.372	0.131	8.06	0.005	1.45	1.12–1.88
BMI (kg/m^2^)	0.049	0.024	4.17	0.041	1.05	1.01–1.09
TC (mmol/L)	0.166	0.061	7.41	0.006	1.18	1.05–1.33
HDL-C (mmol/L)	−1.180	0.478	6.09	0.014	0.31	0.120–0.785

Model also adjusted for TG and LDL-C (both *P* > 0.05, not shown).

Model also adjusted for SBP, TG, and LDL-C (all *P* > 0.05, not shown). All coefficients, standard errors, and confidence intervals have been recalculated and verified; transcription errors in the original version (e.g., direction of male sex, HDL-C interval, age OR) have been corrected.

## 4 Discussion

In this community-based cross-sectional study, we systematically delineated the clinical characteristics and the prevalence of subclinical carotid atherosclerosis (SCAS) across a spectrum of glucose metabolism states in a middle-aged and elderly population. Our data revealed that 55.2% of the screened cohort exhibited abnormal glucose metabolism (including 40.7% with prediabetes and 14.5% with new-onset diabetes). This combined prevalence is notably higher than the reported national average of 35.2% for prediabetes and 12.8% for diabetes (7), which may reflect the increasing cardiometabolic burden driven by rapid urbanization, sedentary lifestyles, and higher living standards in Beijing. Consistent with previous large-scale epidemiological observations (17), we found that classic cardiovascular risk factors, such as age, body weight, blood pressure, lipid profiles, and serum uric acid, deteriorated progressively alongside the exacerbation of glucose dysregulation. Notably, the SCAS detection rate escalated in a stepwise manner from normal glucose tolerance (NGT, 62.2%) to prediabetes (66.8%) and culminated in new-onset diabetes (DM, 70.5%). This stepwise escalation reinforces the concept that atherosclerosis is not a sudden event but a continuous, insidious process, underscoring a strong parallel between the progressive severity of dysglycemia and the accumulating burden of early vascular lesions.

It is widely established that type 2 diabetes serves as a coronary heart disease equivalent. However, the exact cardiovascular prognostic value of the prediabetic stage remains a subject of intense academic debate. Longitudinal studies, including the DECODE study (18) and the Chinese 4C study (17), have demonstrated that IGT is a robust predictor of increased cardiovascular mortality, whereas the predictive utility of isolated IFG is less apparent. Subsequent meta-analyses have reinforced that prediabetes is significantly associated with an elevated risk of all-cause mortality and cardiovascular disease (13), and stroke registries have further highlighted that patients with dysglycemia face higher 1-year mortality and severe disability rates following an ischemic event compared to normoglycemic individuals (19). Regarding subclinical lesions, some reports identify prediabetes as an independent risk factor for SCAS (20). In our multivariable analyses (both in the whole cohort and in the prediabetic subgroup), prediabetes—particularly the IGT phenotype—remained independently associated with SCAS even after adjusting for traditional confounders including age, sex, blood pressure, obesity, and lipids. The strict exclusion of patients with established cardiovascular diseases or those receiving statins and antihypertensive therapies in our study design minimizes pharmacological confounding. This rigorous selection criterion provides a more authentic reflection of the natural atherogenic impact of metabolic clustering in prediabetic individuals, devoid of the plaque-stabilizing effects typically induced by lipid-lowering agents.

A pivotal finding of our subgroup analysis was that the IGT population exhibited a significantly higher prevalence of SCAS and a higher positivity rate for Frank's sign [a recognized clinical surrogate for coronary atherosclerosis (21)] compared to the IFG population. Biologically, this discrepancy may be attributed to the fact that IGT is characterized by severe peripheral insulin resistance and pronounced postprandial glycemic excursions. These sharp spikes in blood glucose may aggressively trigger acute oxidative stress, may induce the production of advanced glycation end products, and may promote endothelial nitric oxide synthase uncoupling. This hostile biochemical environment may subsequently activates inflammatory cascades within the vascular wall to drive premature atherogenesis and structural remodeling (20). Notably, we included the combined IFG + IGT group in our analysis and found that its SCAS burden (81.3%) was the highest among all subgroups, exceeding even the DM group. This unexpected finding warrants cautious interpretation; the unusually high SCAS prevalence in this group, coupled with the observed inconsistency between the composite SCAS endpoint and individual segmental abnormality rates (Table 4), suggests the need for further validation in independent cohorts. Potential data quality issues in this subgroup cannot be entirely excluded and are further discussed in the limitations.

However, in addressing the controversy surrounding the differential vascular risks between IGT and IFG, our findings must be interpreted with caution. Given the inherent limitations of a cross-sectional design, we can only infer a robust co-occurrence or association between the IGT state and increased SCAS prevalence, rather than establish a definitive causal relationship. Crucially, as highlighted in our baseline comparisons, the IFG and IGT subgroups inherently differed in several confounding anthropometric parameters; the IFG group presented with significantly higher body weight, waist circumference, and waist-to-height ratio than the IGT group. Consequently, the extent to which the higher SCAS prevalence in the IGT group is driven purely by postprandial glucose fluctuations, vs. the complex clustering effects of age, central obesity, and blood pressure, remains challenging to fully disentangle. Furthermore, the crude comparison showing higher SCAS detection in NGT (62.2%) than in IFG (55.8%) appears counterintuitive, given that the IFG group had higher age, BMI, waist circumference, SBP, FBG, and TC than the NGT group. The IFG group had a much lower proportion of males (19.3%) compared with NGT (29.4%), and male sex is a strong risk factor (OR = 1.35 in our fully adjusted model). However, based on the sex-specific SCAS rates estimated from our data (approximately 60.2% in females and 67.1% in males), the difference in male proportion between the two groups would explain only about 0.7 percentage points of the 6.4 percentage-point gap, leaving most of the difference unexplained. Thus, the lower SCAS prevalence in IFG vs. NGT cannot be fully attributed to sex composition alone. Given that our multivariable models in Table 5 did not include a direct NGT vs. isolated IFG comparison (Model A combined all prediabetes phenotypes into one category, and Model B used IFG as the reference but excluded NGT), we are unable to determine whether this difference persists after full adjustment. We recommend that future studies with larger sample sizes perform a five-category exposure model (NGT, isolated IFG, isolated IGT, combined IFG + IGT, and DM) with NGT as the common reference to directly address this question. While our multivariable model accounted for these traditional risk factors, future prospective cohort studies with rigorous propensity score matching or adjusting for time-varying covariates are warranted to confirm whether isolated IGT independently dictates a steeper trajectory of carotid atherosclerosis.

Several other critical limitations of this study must be transparently acknowledged. First, the inherent cross-sectional design fundamentally restricts our ability to track longitudinal outcomes or definitively establish a temporal sequence between the onset of dysglycemia and the initiation of carotid plaque formation. It is also susceptible to survival bias, where individuals with the most severe metabolic or vascular conditions might have been excluded due to prior cardiovascular events. Second, our reliance on Frank's sign as a clinical proxy for coronary atherosclerosis, while historically validated in certain cohorts, remains a rudimentary and somewhat subjective physical assessment. The diagonal earlobe crease is thought to reflect systemic loss of dermal and vascular elastic fibers, yet it lacks the diagnostic precision of advanced imaging modalities. Future investigations should incorporate coronary computed tomography angiography (CCTA) or intravascular ultrasound to provide a more precise, quantitative anatomical assessment of the coronary tree. Third, our study population was exclusively drawn from specific community centers in Shijingshan District, Beijing. While this provides a homogenous cohort that minimizes wide sociocultural variations, it inherently limits the generalizability of our findings to rural populations, different geographic regions, or other ethnic groups with distinct genetic backgrounds and dietary patterns. Fourth, the categorization of glucose metabolism statuses was based on a single baseline oral glucose tolerance test (OGTT). Although the OGTT remains the gold standard, intra-individual glycemic variability exists, and the lack of a confirmatory second test might have led to potential misclassification of a small subset of borderline participants. Fifth, despite our rigorous exclusion criteria and multivariable adjustments, the possibility of residual confounding from unmeasured variables cannot be entirely eliminated. Factors such as detailed dietary habits, daily physical activity levels, psychosocial stress, obstructive sleep apnea, and specific genetic predispositions were not comprehensively quantified in this current dataset. In addition, inflammatory biomarkers such as high-sensitivity C-reactive protein (hs-CRP) were not measured, precluding an assessment of the inflammatory pathways that may link dysglycemia to atherosclerosis. In particular, important lifestyle factors including smoking, alcohol consumption, and physical activity were not systematically recorded in this dataset, and therefore none of our models were adjusted for these variables; this may have introduced residual confounding. We acknowledge this as a major limitation of the present analysis. Sixth, we performed sex-stratified sensitivity analyses; the main effect estimates for glucose categories on SCAS were similar in direction and magnitude between sexes (data not shown). However, these stratified results are not presented in detail in the main tables due to space constraints; they are available from the corresponding author upon request.

Despite these limitations, our findings emphasize that the prediabetic state is not merely an isolated glycemic marker, but a profound clustering of metabolic abnormalities that warrants aggressive, multifaceted, and personalized clinical intervention (22).

## 5 Conclusion

This community-based study demonstrates that subclinical carotid atherosclerosis is highly prevalent among middle-aged and elderly individuals with abnormal glucose metabolism. Both new-onset diabetes and prediabetes, particularly the impaired glucose tolerance (IGT) phenotype, are closely associated with an elevated burden of SCAS and a clustering of conventional metabolic risk factors, independent of the measured conventional risk factors. These results provide a rationale for evaluating early detection and comprehensive risk factor management in individuals with dysglycemia in future prospective studies. However, prospective cohort studies are needed to establish causal relationships and to determine whether targeting IGT specifically reduces cardiovascular events.

## Data Availability

The original contributions presented in the study are included in the article/Supplementary Material, further inquiries can be directed to the corresponding author/s.

## References

[1] Liao J Wang L Duan L Gong F Zhu H Pan H. Association between estimated glucose disposal rate and cardiovascular diseases in patients with diabetes or prediabetes: a cross-sectional study. Cardiovasc Diabetol. (2025) 24(1):13. 10.1186/s12933-024-02570-y 39806389 PMC11730478

[2] Thiab S Akhal T Akeblersane M Sheth H Atkin SL Butler AE. Microvascular complications in prediabetes: a systematic review & meta-analysis. Diabetes Res Clin Pract. (2025) 225:112261. 10.1016/j.diabres.2025.112261 40419194

[3] Elzinga SE Guo K Turfah A Henn RE Webber-Davis IF Hayes JM. Metabolic stress and age drive inflammation and cognitive decline in mice and humans. Alzheimers Dement. (2025) 21(3):e70060. 10.1002/alz.70060 40110679 PMC11923576

[4] Dove A Wang J Huang H Dunk MM Sakakibara S Guitart-Masip M. Diabetes, prediabetes, and brain aging: the role of healthy lifestyle. Diabetes Care. (2024) 47(10):1794–802. 10.2337/dc24-0860 39193914 PMC11417282

[5] Cai X Zhang Y Li M Wu JH Mai L Li J. Association between prediabetes and risk of all cause mortality and cardiovascular disease: updated meta-analysis. Br Med J. (2020) 370:m2297. 10.1136/bmj.m2297 PMC7362233 32669282

[6] ElSayed NA McCoy RG Aleppo G Balapattabi K Beverly EA Briggs Early K. American Diabetes Association Professional Practice Committee. 2. Diagnosis and classification of diabetes: standards of care in diabetes-2025. Diabetes Care. (2025) 48(Suppl 1):S27–49. 10.2337/dc25-S002 39651986 PMC11635041

[7] Li Y Teng D Shi X Qin G Qin Y Quan H. Prevalence of diabetes recorded in mainland China using 2018 diagnostic criteria from the American diabetes association: national cross sectional study. Br Med J. (2020) 369:m997. 10.1136/bmj.m997 PMC7186854 32345662

[8] Rooney MR Rawlings AM Pankow JS Echouffo Tcheugui JB Coresh J Sharrett AR. Risk of progression to diabetes among older adults with prediabetes. JAMA Intern Med. (2021) 181(4):511–9. 10.1001/jamainternmed.2020.8774; Erratum in: *JAMA Intern Med* (2021) **181**(4):570. doi: 10.1001/jamainternmed.2021.1321. 33555311 PMC7871207

[9] Sánchez E Betriu À López-Cano C Hernández M Fernández E Purroy F. ILERVAS project collaborators. Characteristics of atheromatosis in the prediabetes stage: a cross-sectional investigation of the ILERVAS project. Cardiovasc Diabetol. (2019) 18(1):154. 10.1186/s12933-019-0962-6 PMC6857207 31729979

[10] Johri AM Hill B Grubic N Sirwani B Fraser M Hétu MF. The prevalence of carotid subclinical atherosclerosis according to age: a systematic review and meta-analysis of the young to middle-age. J Clin Lipidol. (2026) 20(2):250–61. 10.1016/j.jacl.2025.10.069 41449009

[11] Shimoda S Kitamura A Imano H Cui R Muraki I Yamagishi K. Associations of carotid intima-media thickness and plaque heterogeneity with the risks of stroke subtypes and coronary artery disease in the Japanese general population: the circulatory risk in communities study. J Am Heart Assoc. (2020) 9(19):e017020. 10.1161/JAHA.120.017020 32990124 PMC7792402

[12] Vilanova MB Franch-Nadal J Falguera M Marsal JR Canivell S Rubinat E. Prediabetes is independently associated with subclinical carotid atherosclerosis: an observational study in a non-urban Mediterranean population. J Clin Med. (2020) 9(7):2139. 10.3390/jcm9072139 32645918 PMC7408832

[13] Huang Y Cai X Mai W Li M Hu Y. Association between prediabetes and risk of cardiovascular disease and all cause mortality: systematic review and meta-analysis. Br Med J. (2016) 355:i5953. 10.1136/bmj.i5953 PMC5121106 27881363

[14] Stoyanov GS Dzhenkov D Petkova L Sapundzhiev N Georgiev S. The histological basis of frank’s sign. Head Neck Pathol. (2021) 15(2):402–7. 10.1007/s12105-020-01205-4 32712879 PMC8134579

[15] Zhang L-G Li H-J Liu S Liu J-Y. Correlation between the TyG-BMI index and carotid plaque characteristics in middle-aged and elderly patients with acute myocardial infarction. Clin Hemorheol Microcirc. (2024) 88(4):455–62. 10.3233/CH-242354 39121115

[16] Montenegro-González GC Bea C Ampudia-Blasco FJ González-Navarro H Real JT Peñarrocha-Diago M. Usefulness of the CDC/AAP and the EFP/AAP criteria to detect subclinical atherosclerosis in subjects with diabetes and severe periodontal disease. Diagnostics. (2025) 15(7):928. 10.3390/diagnostics15070928 PMC11988492 40218278

[17] Jia X Wang S Wang J Ding Y Li M Lin Y. 4C study author. Data-driven phenotypic profiling of prediabetes reveals heterogeneous cardiometabolic risks in Chinese adults. Cardiovasc Diabetol. (2026) 25(1):3. 10.1186/s12933-025-03008-9 PMC12777505 41331573

[18] Ning F Tuomilehto J Pyörälä K Onat A Söderberg S Qiao Q. DECODE Study Group. Cardiovascular disease mortality in europeans in relation to fasting and 2-h plasma glucose levels within a normoglycemic range. Diabetes Care. (2010) 33(10):2211–6. 10.2337/dc09-2328 20424221 PMC2945162

[19] Wu S Wang C Jia Q Liu G Hoff K Wang X. Hba1c is associated with increased all-cause mortality in the first year after acute ischemic stroke. Neurol Res. (2014) 36(5):444–52. 10.1179/1743132814Y.0000000355 24649851

[20] Cao Q Xin Z He R Wang T Xu M Lu J. Age-specific difference in the association between prediabetes and subclinical atherosclerosis: an analysis of a Chinese prospective cohort study. Cardiovasc Diabetol. (2022) 21(1):153. 10.1186/s12933-022-01592-8 35948892 PMC9364510

[21] Gakovic B Neskovic SA Vranic I Grujicic K Mijatovic S Ljubojevic A. The relationship of diagonal earlobe crease (frank’s sign) and obstructive coronary artery disease in patients undergoing coronary angiography. Wien Klin Wochenschr. (2023) 135(23–24):667–73. 10.1007/s00508-023-02297-y 37902857

[22] Knowler WC Barrett-Connor E Fowler SE. Reduction in the incidence of type 2 diabetes with lifestyle intervention or metformin. N Engl J Med. (2002) 346(6):393–403. 10.1056/NEJMoa012512 11832527 PMC1370926

