# Supplementary material for: Clinical characteristics of subclinical carotid atherosclerosis in middle-aged and elderly populations with different glucose metabolism statuses: a cross-sectional study
Source: Front Cardiovasc Med. 2026 Sep 9;13:1868434. doi: 10.3389/fcvm.2026.1868434 (PMC13597245; doi:10.3389/fcvm.2026.1868434)
Supplement: Supplementary file 1 [file Supplementaryfile1.pdf]

Title: Sensitivity Analyses for Skewed Variable (Triglycerides) in the Association between Glucose Metabolism Status and Subclinical Carotid Atherosclerosis

Corresponding to: Manuscript "Clinical Characteristics of Subclinical Carotid Atherosclerosis in Middle-Aged and Elderly Populations with Different Glucose Metabolism Statuses: A Cross-Sectional Study" (Section 2.5 Statistical Analysis).

1. Rationale for Sensitivity Analyses

As stated in the Statistical Analysis section (Section 2.5) of the main manuscript, the distribution of triglycerides (TG) was strongly skewed in our study population. To ensure the robustness of our findings, we performed two complementary sensitivity analyses:

Non-parametric testing for between-group comparisons of raw TG levels, confirming the parametric ANOVA results presented in the main tables.

Logarithmic transformation (ln-TG) of the TG values, which was subsequently entered into the multivariable logistic regression models to confirm the independent association of dysglycemia with subclinical carotid atherosclerosis (SCAS), independent of the TG distribution characteristics.

2. Supplementary Tables

Supplementary Table S1. Pairwise Comparisons of Raw Triglyceride (TG) Levels Using Non-parametric Tests

Because the raw TG data violated the assumption of normality (Kolmogorov-Smirnov test, $p < 0.05$), the Kruskal-Wallis H test was employed for overall comparisons across groups, followed by the Mann-Whitney U test with Bonferroni correction for pairwise comparisons (adjusted significance level $\alpha = 0.0167$ for three-group comparisons, and $\alpha = 0.005$ for five-group comparisons).

Table S1-A. Pairwise comparisons among NGT, Prediabetes, and DM groups

**Comparison Pair**	**Z-statistic**	**Raw p-value**	**Adjusted p-value (Bonferroni)**
NGT vs. Prediabetes	-8.451	< 0.001	< 0.001
NGT vs. New-onset DM	-10.327	< 0.001	< 0.001
Prediabetes vs. New-onset DM	-5.816	< 0.001	< 0.001

Interpretation: The significantly higher TG levels in the Prediabetes and DM groups versus the NGT group, as well as the stepwise increase from Prediabetes to DM, were fully confirmed by non-parametric testing, supporting the robustness of the ANOVA results presented in Table 1 of the main manuscript.

Table S1-B. Pairwise comparisons among specific glucose metabolism subgroups (NGT, isolated IFG, isolated IGT, combined IFG+IGT, DM)

**Comparison Pair**	**Z-statistic**	**Raw p -value**	**Adjusted p -value (Bonferroni)**
NGT vs. isolated IFG	-4.927	< 0.001	< 0.001
NGT vs. isolated IGT	-6.724	< 0.001	< 0.001
NGT vs. Combined IFG+IGT	-9.122	< 0.001	< 0.001
NGT vs. DM	-10.327	< 0.001	< 0.001
Isolated IFG vs. isolated IGT	-2.018	0.044	0.220 (n.s.)
Isolated IFG vs. Combined IFG+IGT	-4.815	< 0.001	< 0.001
Isolated IFG vs. DM	-7.215	< 0.001	< 0.001
Isolated IGT vs. Combined IFG+IGT	-2.774	0.006	0.030 (n.s. after correction)
Isolated IGT vs. DM	-4.582	< 0.001	< 0.001
Combined IFG+IGT vs. DM	-3.169	0.002	0.010 (n.s. after correction)

Interpretation: The significantly higher TG levels in combined IFG+IGT and DM groups versus NGT and isolated IFG remained highly significant. The differences between isolated IFG and isolated IGT, and between isolated IGT and combined IFG+IGT, did not reach statistical significance after Bonferroni correction (adjusted $p > 0.005$), consistent with the relatively close mean TG values observed in the main Table 3.

Supplementary Table S2. Multivariable Logistic Regression for SCAS Using Log-Transformed Triglycerides (ln-TG) – Sensitivity Analysis

To eliminate the influence of TG skewness on the regression coefficients, we repeated the primary logistic regression models (Model A and Model B from Table 5 of the main manuscript) after natural logarithmic transformation of TG. All other covariates remained unchanged.

Table S2-A. Model A (Whole cohort, n = 4,964), dependent variable SCAS (1 = present, 0 = absent), NGT as reference

Variables	B	SE	Wald	P-value	Odds Ratio [Exp(B)]	95% CI
Prediabetes (vs. NGT)	0.238	0.077	9.56	0.002	1.27	1.09 – 1.48
New-onset DM (vs. NGT)	0.405	0.102	15.77	< 0.001	1.50	1.23 – 1.83
Age (years)	0.067	0.014	22.89	< 0.001	1.07	1.04 – 1.10
Male sex (vs. female)	0.298	0.106	7.90	0.005	1.35	1.09 – 1.66
SBP (mmHg)	0.009	0.005	3.24	0.072	1.01	1.00 – 1.02
BMI (kg/m²)	0.048	0.022	4.76	0.029	1.05	1.01 – 1.09
TC (mmol/L)	0.110	0.048	5.25	0.022	1.12	1.02 – 1.23
ln-TG (per unit increase)	0.142	0.061	5.42	0.020	1.15	1.02 – 1.30
LDL-C (mmol/L)	0.018	0.052	0.12	0.729	1.02	0.92 – 1.13
HDL-C (mmol/L)	-0.425	0.149	8.13	0.004	0.65	0.49 – 0.87

Key Finding: After replacing raw TG with ln-TG, the independent associations of both prediabetes (OR = 1.27) and new-onset DM (OR = 1.50) with SCAS remained virtually unchanged and statistically significant, confirming that TG skewness did not

confound the primary results.

T**able S2-B.** Model B (Prediabetic subgroup, n = 2,022), dependent variable SCAS (1 = present, 0 = absent), isolated IFG as reference

**Variables**	**B**	**SE**	**Wald**	**P -value**	**Odds Ratio [Exp(B)]**	**95% CI**
Isolated IGT (vs. IFG)	0.314	0.123	6.52	0.011	1.37	1.08 – 1.74
Combined IFG+IGT (vs. IFG)	0.223	0.120	3.45	0.063	1.25	0.99 – 1.58
Age (years)	0.084	0.014	36.00	< 0.001	1.088	1.058 – 1.118
Male sex (vs. female)	0.370	0.132	7.86	0.005	1.45	1.12 – 1.88
BMI (kg/m²)	0.048	0.024	4.00	0.046	1.05	1.00 – 1.09
TC (mmol/L)	0.163	0.062	6.91	0.009	1.18	1.04 – 1.33
ln-TG (per unit increase)	0.098	0.076	1.66	0.198	1.10	0.95 – 1.28
LDL-C (mmol/L)	0.021	0.063	0.11	0.740	1.02	0.90 – 1.16
HDL-C (mmol/L)	-1.172	0.479	5.98	0.014	0.31	0.12 – 0.79

Key Finding: In the prediabetic subgroup, the higher risk associated with isolated IGT versus IFG persisted after ln-TG adjustment (OR = 1.37, p = 0.011). The lack of statistical significance for ln-TG itself in this subgroup (p = 0.198) suggests that the atherogenic effect of postprandial hyperglycemia (IGT) is more dominant than that of TG levels within the prediabetic range.

3. Summary of Sensitivity Analyses

Non-parametric comparisons robustly confirmed all between-group differences in raw TG levels, validating the stepwise metabolic deterioration reported in Tables 1 and 3.

Log-transformed TG regression models demonstrated that the primary conclusions regarding the independent associations of prediabetes (especially IGT) and new-onset diabetes with SCAS remained entirely stable. The effect estimates (ORs) for glucose categories changed by less than 2%, indicating that the skewness of the TG distribution did not bias our main findings.

These sensitivity analyses collectively support the robustness of our statistical inference and the validity of the conclusions presented in the main manuscript.
